# Mesenchymal stem cells inhibit T cell activation by releasing TGF-β1 from TGF-β1/GARP complex

**DOI:** 10.18632/oncotarget.21549

**Published:** 2017-10-06

**Authors:** Jian Niu, Wang Yue, Zhu Le-Le, Liu Bin, Xin Hu

**Affiliations:** ^1^ General Surgery of the Hospital Affiliated Hospital of Xuzhou Medical University, Digestive Disease Research Laboratory of Xuzhou Medical University, Xuzhou, Jiangsu 221002, PR China; ^2^ The University of Texas Graduate School of Biomedical Sciences at Houston, MD Anderson Cancer Center, Houston, TX 77030, USA

**Keywords:** glycoprotein A repetitions predominant, mesenchymal stem cells, TGF-β, proliferation, immunomodulation

## Abstract

Intervention with mesenchymal stem cells (MSCs) reveals a promising therapeutic tool to treat transplantation and autoimmune disease due to their immunoregulation capability. But the mechanisms of action are not fully investigated yet. Transforming growth factor-β1 (TGF-β1) exhibit multiple effects in migration, differentiation, and immunomodulation of MSCs. Glycoprotein A repetitions predominant (GARP) is an important marker of activated Treg (regulatory T cells). GARP binds latent TGF-β1 to regulate its activation, which is the indispensable step in Treg suppressing effector T cells. So far we don’t know whether GARP present on MSCs and its association with MSCs function. Our study show that MSCs express GARP which binds latent TGF-β1 on their cell surface. We also found that TGF-β1+/− MSCs produce less TGF-β1 and exhibit reduced capacity in inhibiting T cells. When TGF-β1 signaling pathway was blocked, MSCs show decreased activity in inhibiting T cells.

Importantly, silencing GARP expression distinctively damaged the capacity of MSCs to inhibit IFN-γ production. These findings indicated the expression of GARP on MSCs and its functionality in activating LAP, thus demonstrating GARP as a novel biomarker and new target to improve the therapeutic efficacy of MSCs.

## INTRODUCTION

MSCs have the potential to effectively treat a wide range of diseases. Above all, MSCs are among the first stem cells to be applied into clinical practice [1, 2]. MSCs have immunoregulation capability by both inhibiting the maturation/differentiation of dendritic cells and by suppressing the activation and/or function of T, B and NK cells.

It is certain that we need to find the better characterization of MSCs and better understanding of of MSCs biology.

TGF-β1 plays critical roles in many physiological and pathological conditions, including cell differentiation and apoptosis, inflammation regulation and tissue repair.

First of all, TGF-β1 is produced as pro-TGF-β1 precursor in cells. Then pro-TGF-β1 is cleaved into two fragments [3, 4].

The C-terminal homodimer represents mature TGF-β1 and the N-terminal homodimer is named as LAP (latency-associated peptide). Mature TGF-β1 binds to LAP in one complex called latent TGF-β1.

Latent TGF-β1 is secreted by many cells, such as human Treg [5, 6]. Latent TGF-β1 has no biological activity because LAP prevents mature TGF-β1 to bind to its receptor. Mature TGF-β1 can be released from the LAP to become active. This process is called as TGF-β1 activation.

GARP is identified as the significant marker for activated Treg [7, 8].GARP can anchor and activate latent TGF-β1 on cell surface. The process is required by Tregs to inhibit effector T cells and to induce tolerance via cell-to-cell contact [9–11].

The distribution of GARP is thought to be restricted on platelets and activated Tregs[7]. Little is known about whether GARP is expressed on other cells, and if it is, whether GARP plays any role in regulating the function of these cells.

Recently, scientists found that latent TGF-β1 was expressed on human umbilical cord blood-derived MSCs [12] and on murine bone marrow-derived MSCs [13]. But, the method of TGF-β1 attaching to MSCs and the role of latent TGF-β1 in MSCs functions is not clear.

In this study, we examined the role of TGF-β1 in MSCs-mediated T cell inhibition. Then , we test the expression and function of GARP on primary MSCs. Our results show that GARP is constitutively expressed on MSCs and is required by MSCs to activate locally produced latent TGF-β1, thus inhibiting the proliferation of T cells and inflammatory cytokine production.

## RESULTS

### TGF-β1 is necessary for MSCs to increase T cells function

**Figure 1:**
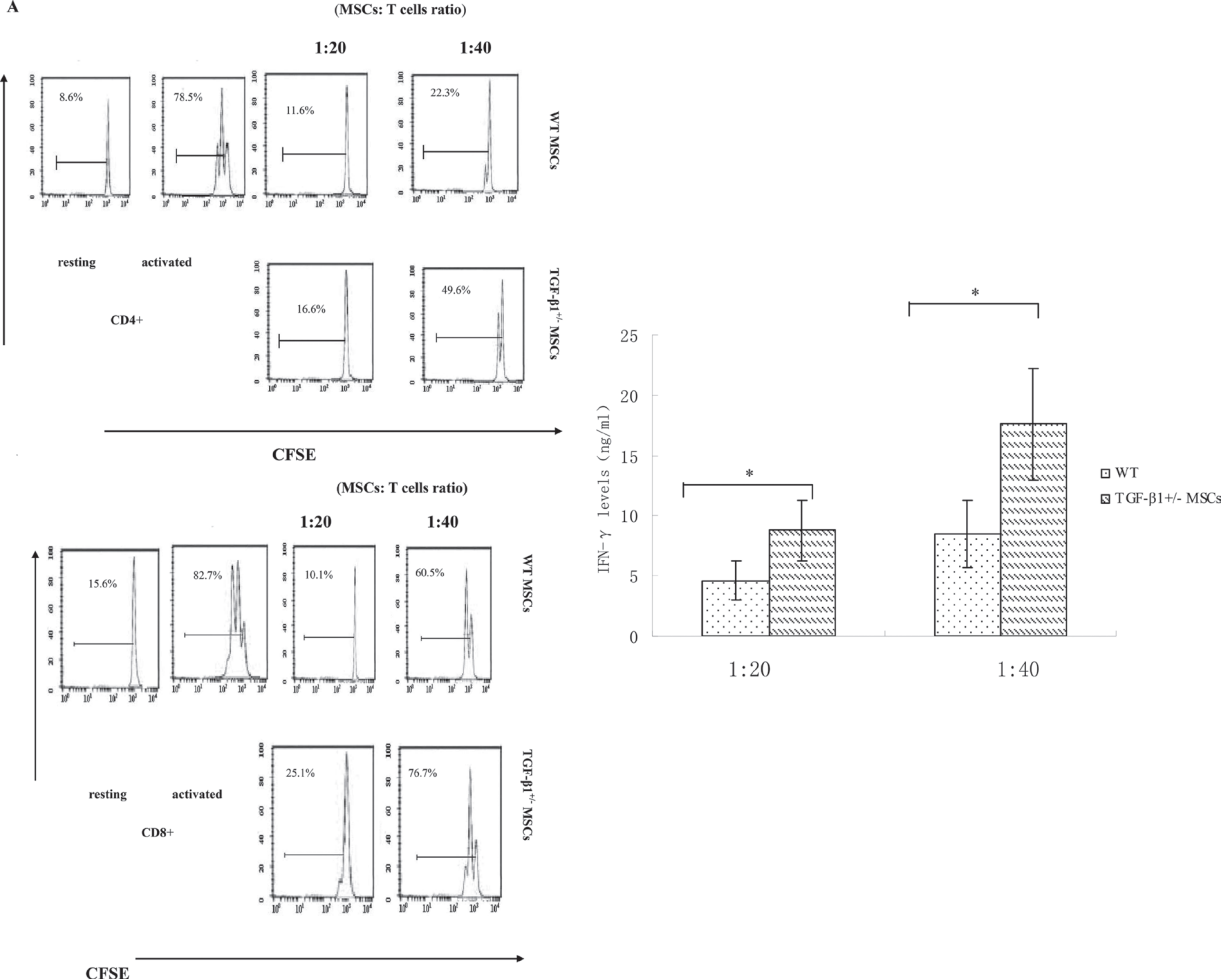

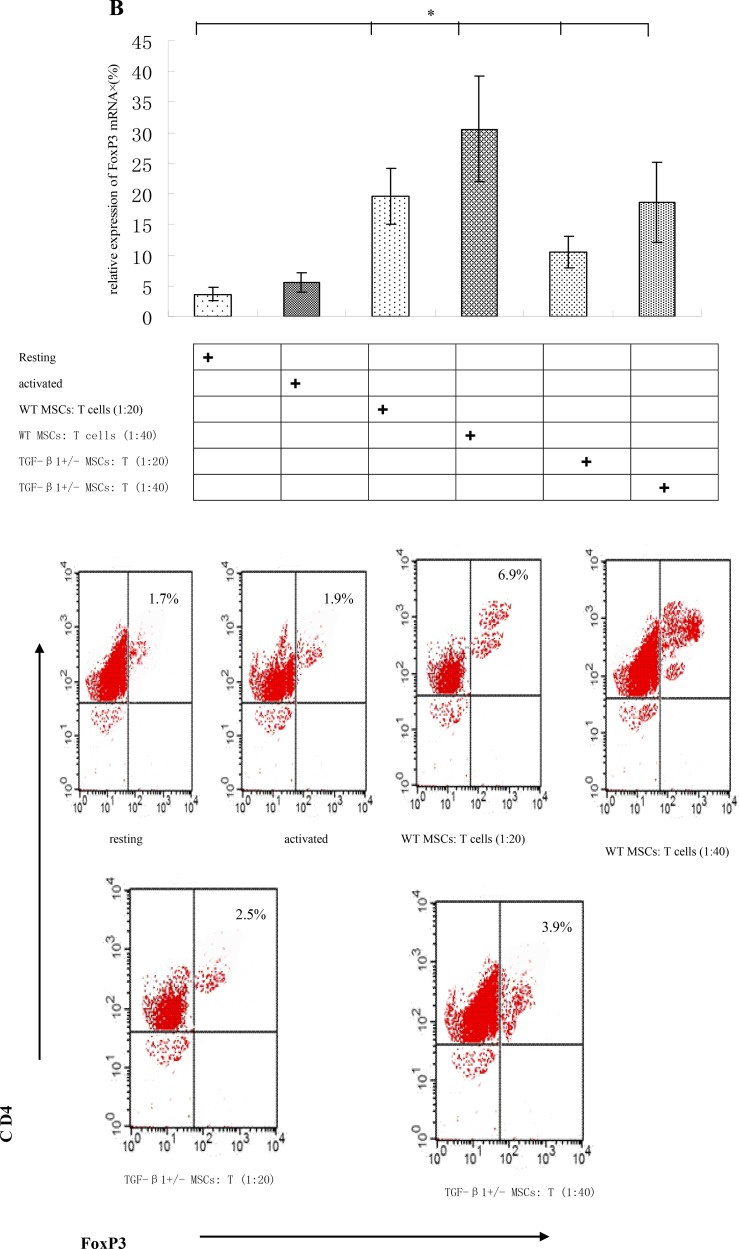

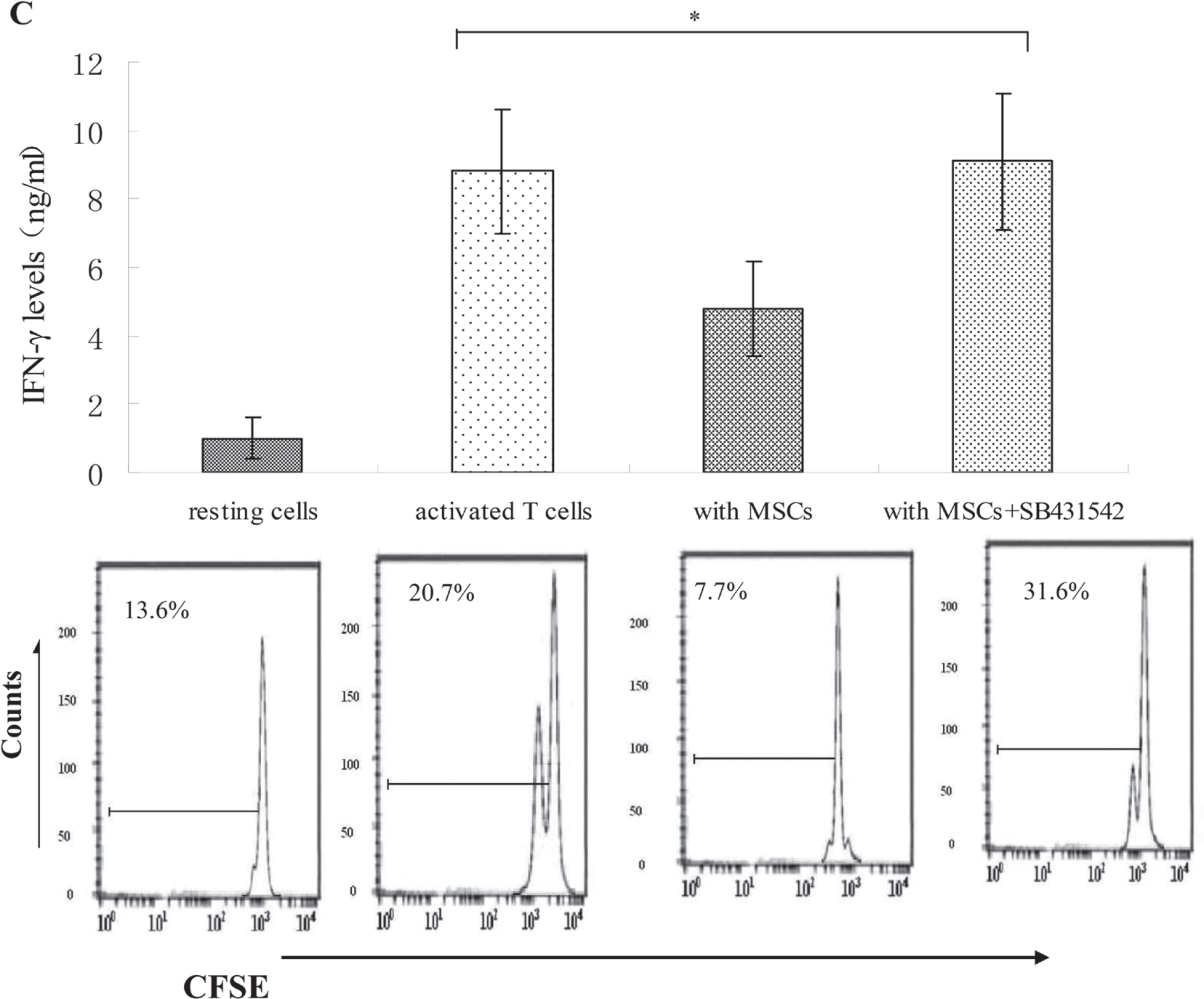
TGF-β1 is required for MSCs to efficiently inhibit T cells (**A**) WT and TGF-β1+/− MSCs were cocultured with CFSE-labeled, anti-CD3/CD28mAb–activated T cells at different ratios (MSCs/T cells). Seventy-two hours later, proliferations of the activated CD4+ and CD8+ T cells were assessed by flow cytometry. Additionally, IFN-γ levels in the culture supernatants were measured by ELISA. Results are representative of three different experiments. ^*^p < 0.05 , vs WT MSCs group. (**B**) WT and TGF-β1+/− MSCs were cocultured with CFSE-labeled, anti-CD3/CD28mAb–activated T cells at different ratios (MSCs/T cells). Seventy-two hours later, FoxP3 mRNA and protein were assessed by quantitative real-time PCR and flow cytometry. ^*^P < 0.05 compared with resting T Dot plots are representative of three independent experiments. The numbers in the upper right quadrants indicate the percentage of double-positive cells. (**C**) WT MSCs were cocultured with CFSE-labeled, anti-CD3/CD28 mAb–activated T cells at a 1:15 ratio in the absence or presence of 2 mM TGF-β1 signaling inhibitor SB431542. Proliferation of the activated T cells and their production of IFN-γ were assessed in 72 h by flow cytometry and ELISA. Results are representative of three different experiments. ^*^p < 0.05 , vs MSCs group.

MSCs are known to be one source of TGF-β1 [14–15], but whether TGF-β1 secreted by MSCs exhibit potent immunosuppressive activity remains unknown. To address this issue, we isolated MSCs from WT or TGF-β1^+/−^ mice. We found TGF-β1^+/−^ MSCs present reduced T cell inhibitory activity (Figure 1A), indicating that TGF-β1 is necessary for MSCs to inhibit T cells.

We also found significant increase of FoxP3 expression in T cells co-cultured in the presence of WT MSCs (Figure 1B). Since mRNA level of FoxP3does not always correlate with its protein levels; we further examine FoxP3 expression using flow cytometry. We observed consistent increase in FoxP3 expression after co-culture with WT MSCs (Figure 1B), suggesting WT MSCs could induce increased FoxP3 expression in allogeneic T cells.

In addition, we inoculated WT MSCs with T cells in the presence of the TGF-β1 signaling inhibitor SB431542 [16] and then measured the proliferation of these T cells. Our data showed that inhibiting TGF-β1 signaling significantly increased the T cells proliferation and IFN-γ production of T cells in the presence of MSCs (Figure 1C), indicating that TGF-β signaling played an important role in MSCs-mediated T cell inhibition.

### TGF-β1 signaling pathway played a crucial role for MSCs to inhibit T cells

**Figure 2:**
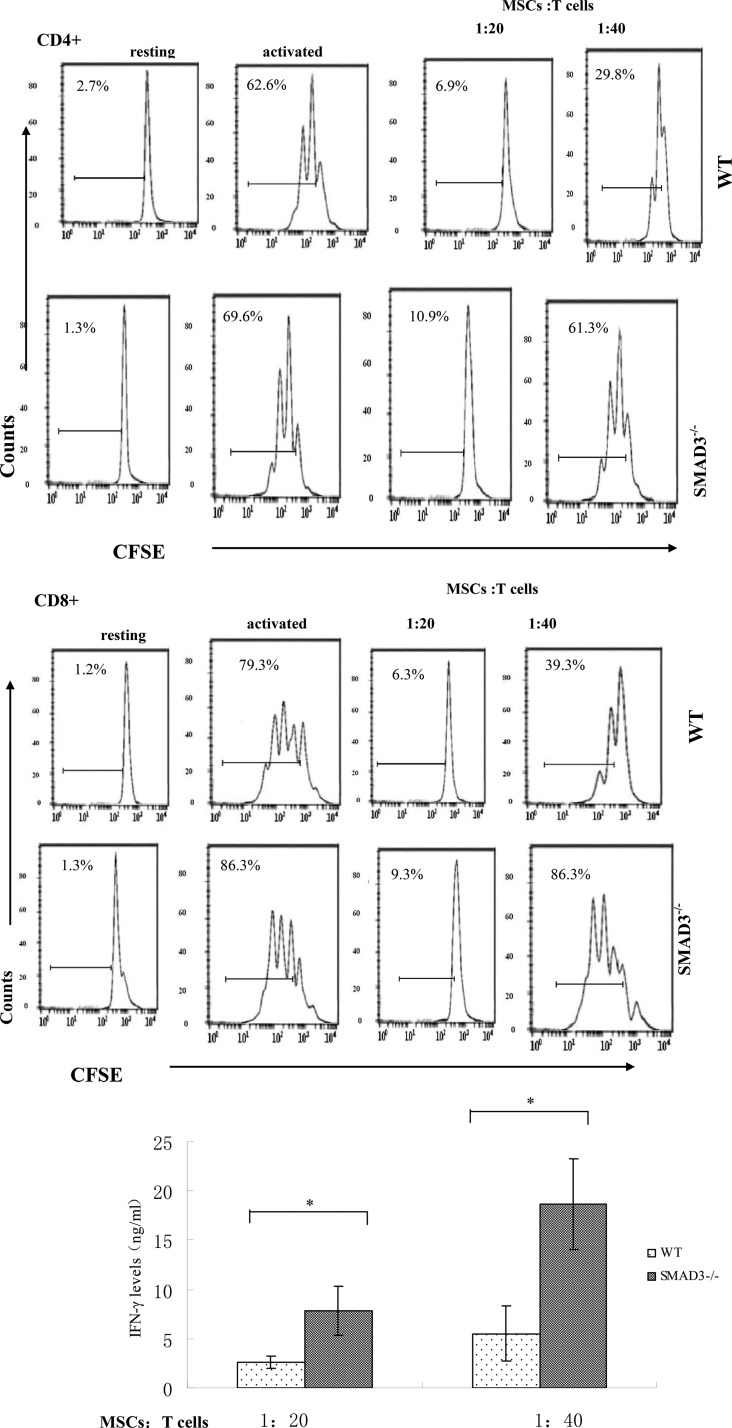
MSCs -produced TGF-β1 directly inhibits T cells through the SMAD3 pathway WT MSCs were cocultured with CFSE-labeled, anti-CD3/CD28 mAb–activated WT or SMAD3−/− T cells at different ratios (MSCs /T cells). Seventy-two hours later, proliferation of the activated T cells was assessed by flow cytometry and production of IFN-γ by the activated T cells was measured by ELISA. Results are representative of three different experiments. ^*^p < 0.05 , vs WT MSCs group.

MSCs-produced latent TGF-β1, which could directly initiate signaling pathways in T cells to exhibit their T cell inhibitory activity upon activation, alternatively it could also regulate MSCs in an autocrine fashion to indirectly inhibit T cells, e.g. by upregulating PD-L1 expression in MSCs. In light of previous reports that SMAD3 is a critical intracellular signal transducer and transcriptional modulator for TGF-β1, we cultured MSCs with different numbers of activated WT and SMAD3^−/−^ T cells and then assessed the proliferation and cytokine production of these T cells. SMAD3^−/−^ T cells showed distinctly increased proliferation and IFN-γ production compared with WT T cells, which were potently suppressed by the MSCs (Figure 2), indicating that MSC-produced TGF-β1 could directly regulate these T cells to inhibit their proliferation and cytokine production and that the SMAD3 pathway of TGF-β1 signaling is important for the TGF-β1 secreting MSCs to inhibit T cells activity. Collectively, these results revealed a previously unknown mechanism of the MSCs -produced TGF-β1 by which MSCs inhibit T cells.

### GARP is expressed on human and mouse MSCs

**Figure 3:**
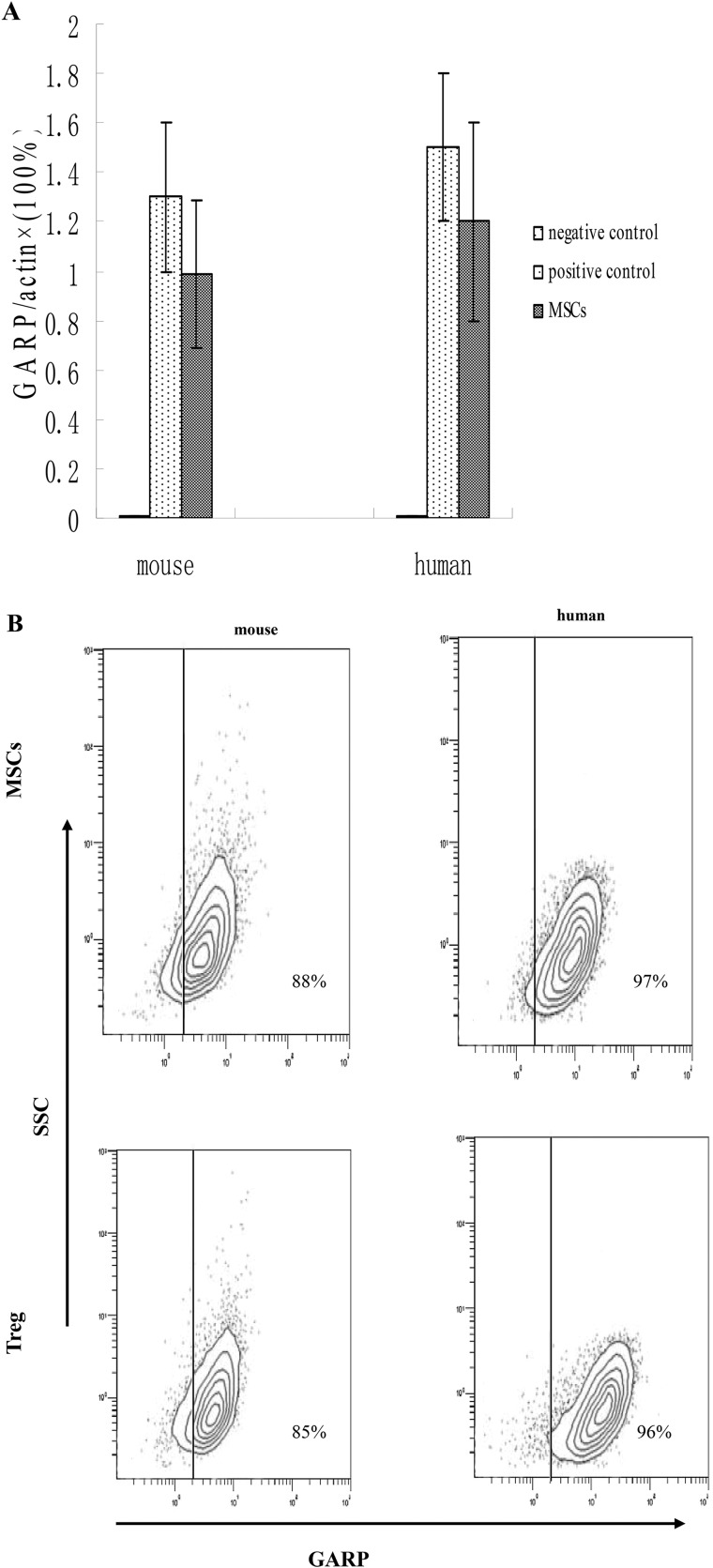
MSCs constitutively express GARP (**A**) GARP were analyzed in mouse and human primary MSCs by qPCR.293T cells as negative control and Treg cells as positive control. (**B**) Both mouse and human MSCs were also analyzed by flow cytometry. Results are representative of three different experiments. Treg cells as positive control.

Previous studies have proven that GARP is necessary for Tregs to activate latent TGF-β1 [17–18]. Thus, we explored whether GARP is also expressed on MSCs and whether it is important for MSCs to inhibit T cells through activating latent TGF-β1. First, we measured GARP expression on MSCs by qPCR. We found that GARP is constitutively transcribed in mouse MSCs and human MSCs. (Figure 3A). To determine the presence of GARP proteins, we measured GARP expression on human and mouse primary MSCs by flow cytometry. Consistent with the qPCR results, GARP proteins were detectable on the cell surface in both human and mouse MSCs (Figure 3B), indicating that GARP is expressed on the cell surface of human and mouse MSCs.

### GARP expression levels are increased on MSCs activated by IFN-γ

**Figure 4:**
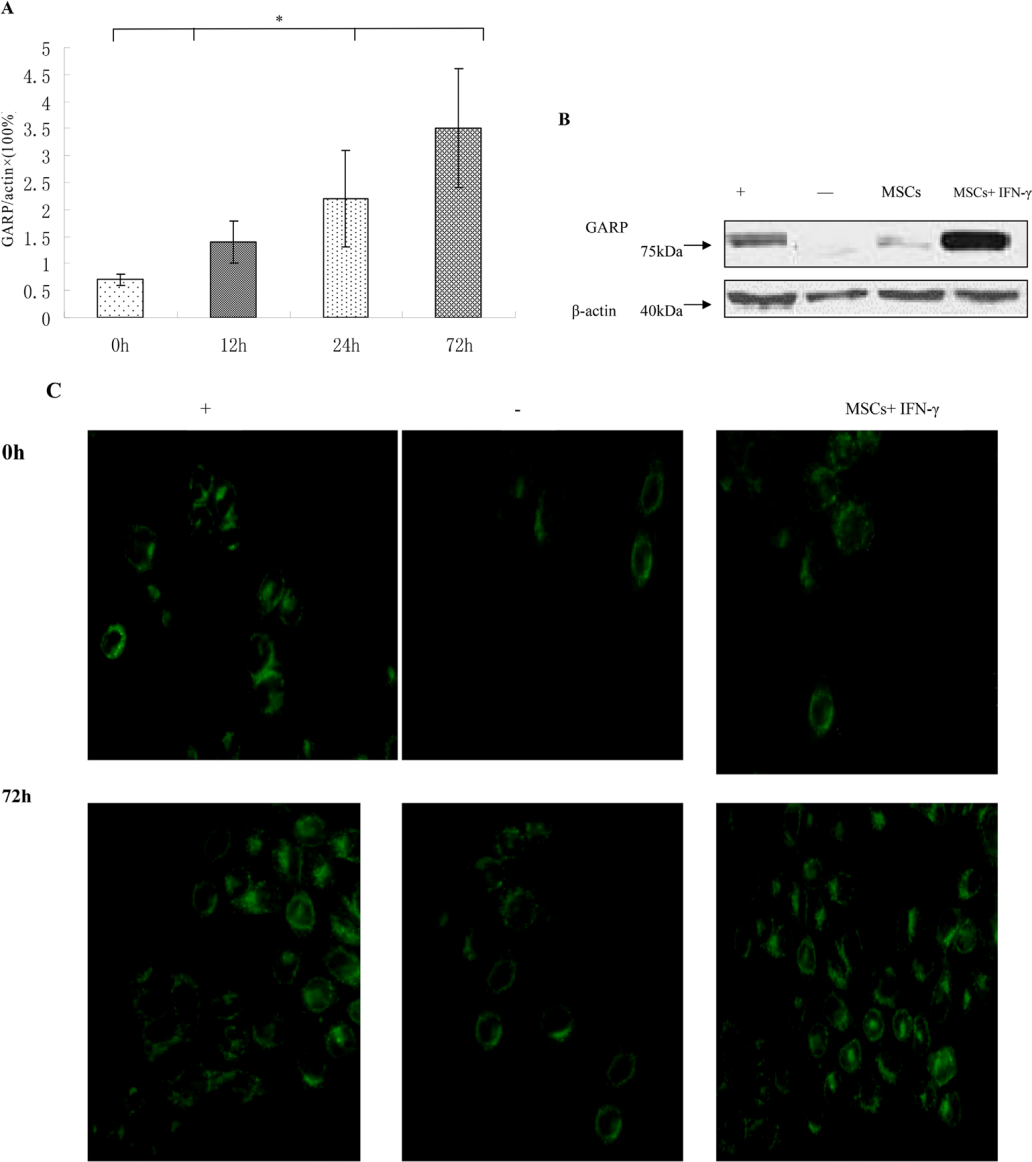
GARP expression is upregulated by IFN-γ (**A**) MSCs were incubated with 100 U/ml IFN-γ and then GARP was assessed by qRT-PCR at different time points. ^*^p < 0.05 , vs 0 h group. (**B**) The expression of GARP was detected by western blot. The production of GARP protein was higher in MSCs+ IFN-γ group than other group. (**C**) immunofluorescence staining (original magnification×400). Results are representative of three different experiments. -, negative control (CD8+ T cells); +, positive control (Treg cells) ; MSCs+IFN-γ (MSCs with IFN-γ stimulation for 72 h).

To evaluate the effect of IFN-γ on GARP expression, we stimulated MSCs with 100 U IFN-γ for 0, 12, 24, or 72 h and then measure mRNA level of GARP. Our data showed that the expression of GARP increased in MSCs stimulated by IFN-γ (Figure 4A). Then we stimulated MSCs with 100 U IFN-γ for 72 h and examined GARP protein levels by Western blots and immunofluorescence staining. Consistent with increased mRNA levels of GARP, GARP protein levels increased on the surface of MSCs after incubation with IFN-γ (Figure 4B–4C), suggesting that IFN-γ could upregulate the expression of GARP on MSCs.

### Breaking GARP function damages the T cell inhibitory activity of MSCs

**Figure 5:**
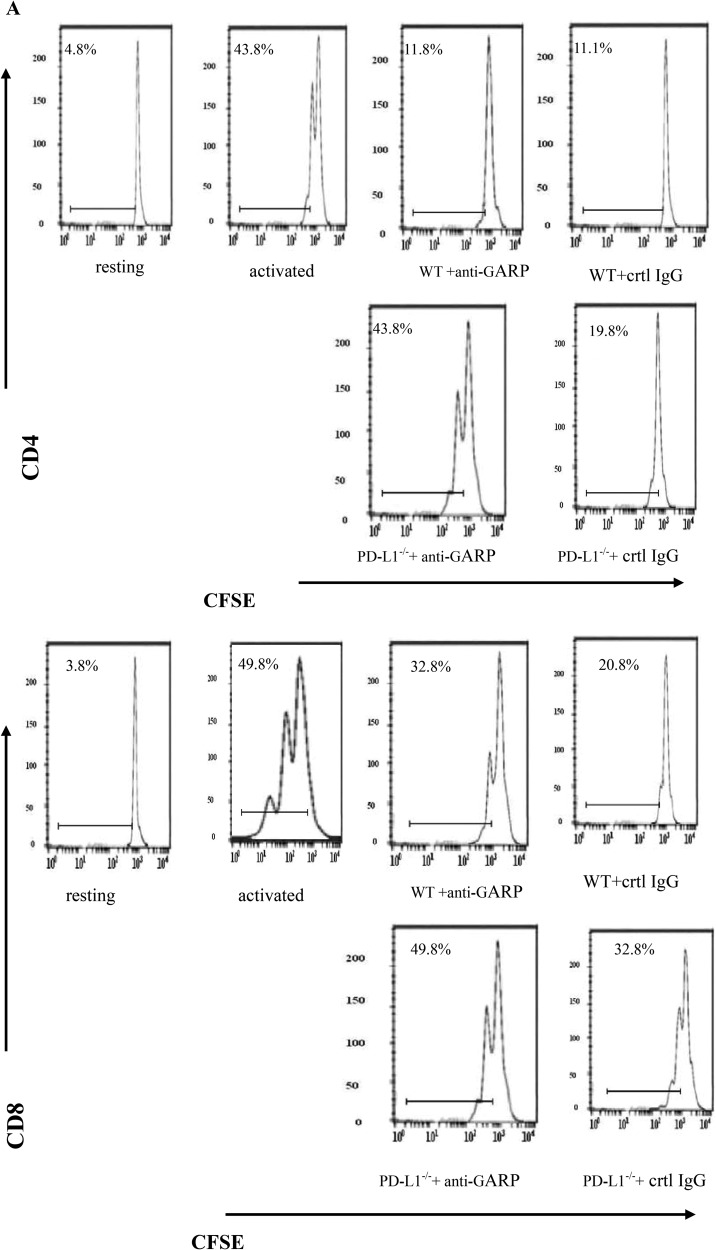

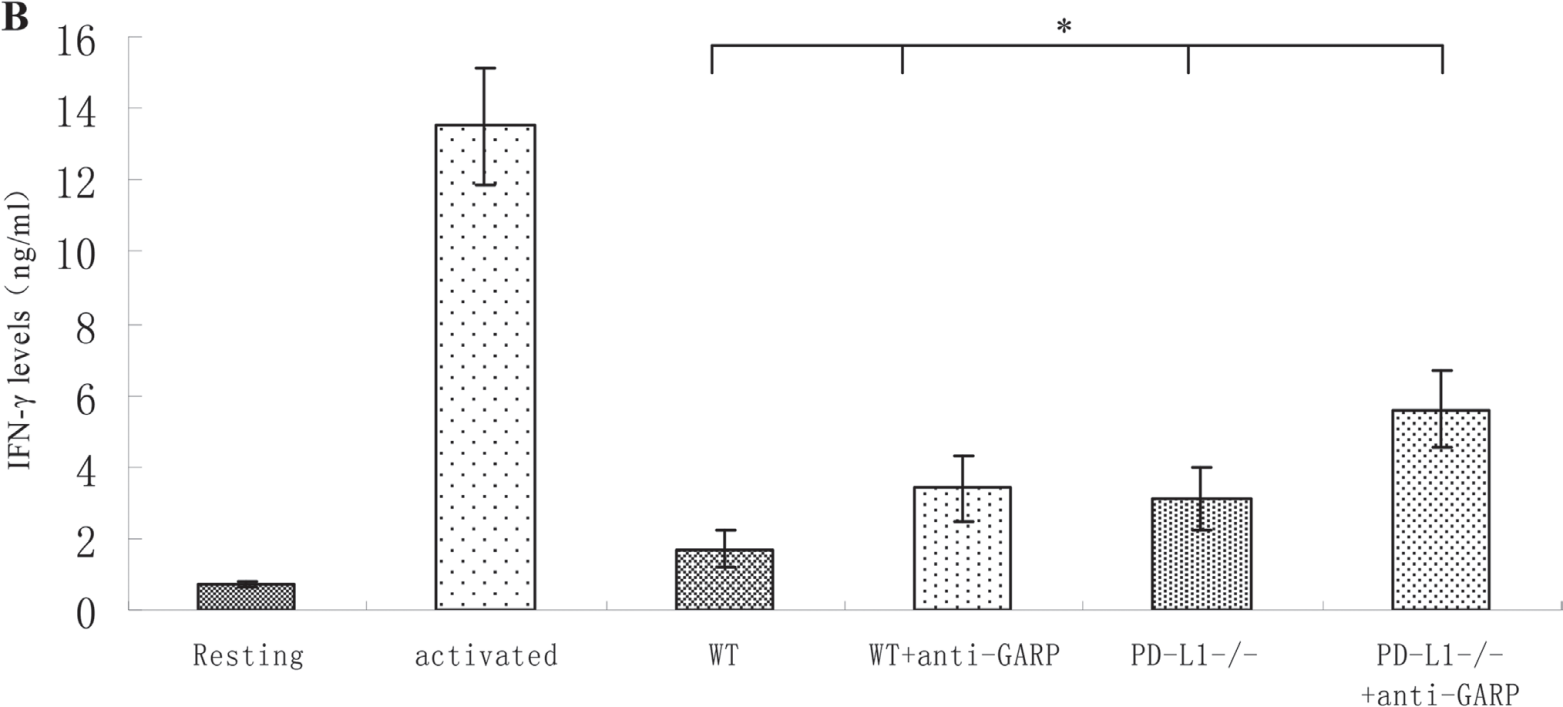
GARP is required for MSCs to efficiently inhibit T cells in addition to PD-L1 WT or PD-L1−/− MSCs were cocultured with CFSE-labeled, anti-CD3/CD28 mAb–activated T cells at 1:20 ratios in the presence of control IgG or 10 mg/ml polyclonal anti-GARP IgGs for seventy-two hours. (**A**) Proliferation of CD4+ and CD8+ T cells was assessed by flowcytometry. (**B**) IFN-γ levels in the culture supernatants were measured by ELISA. Results are representative of three different experiments. ^*^p < 0.05 ^*^p < 0.05 , vs WT group.

Next we examined the role of GARP in MSCs -mediated T cell inhibition and its relevance with PD-L1 expression on MSCs. We incubated PD-L1^−/−^ MSCs with activated T cells in the presence of GARP blocking IgGs for 72 h and then examined the proliferation of T cells. We further measured the levels of secreting IFN-γ in the culture supernatants by ELISA. Our findings are in alignment with previous reports [19–20] that MSCs could remarkably inhibited both T cells proliferation and IFN-γ production. But PD-L1^−/−^ MSCs exhibited reduced T cell inhibitory capacity (Figure 5). In the presence of GARP-blocking IgGs, the inhibitory effect of WT MSCs was also significantly reduced. Interestingly, PD-L1^−/−^ MSCs in the presence of GARP-blocking IgGs showed the weakest T cell inhibitory activity (Figure 5). These results suggest that in addition to PD- L1, GARP could function as another cell surface regulator required by MSCs to inhibit T cells.

### Knocking down of GARP impairs the T cell inhibitory effect of MSCs

**Figure 6:**
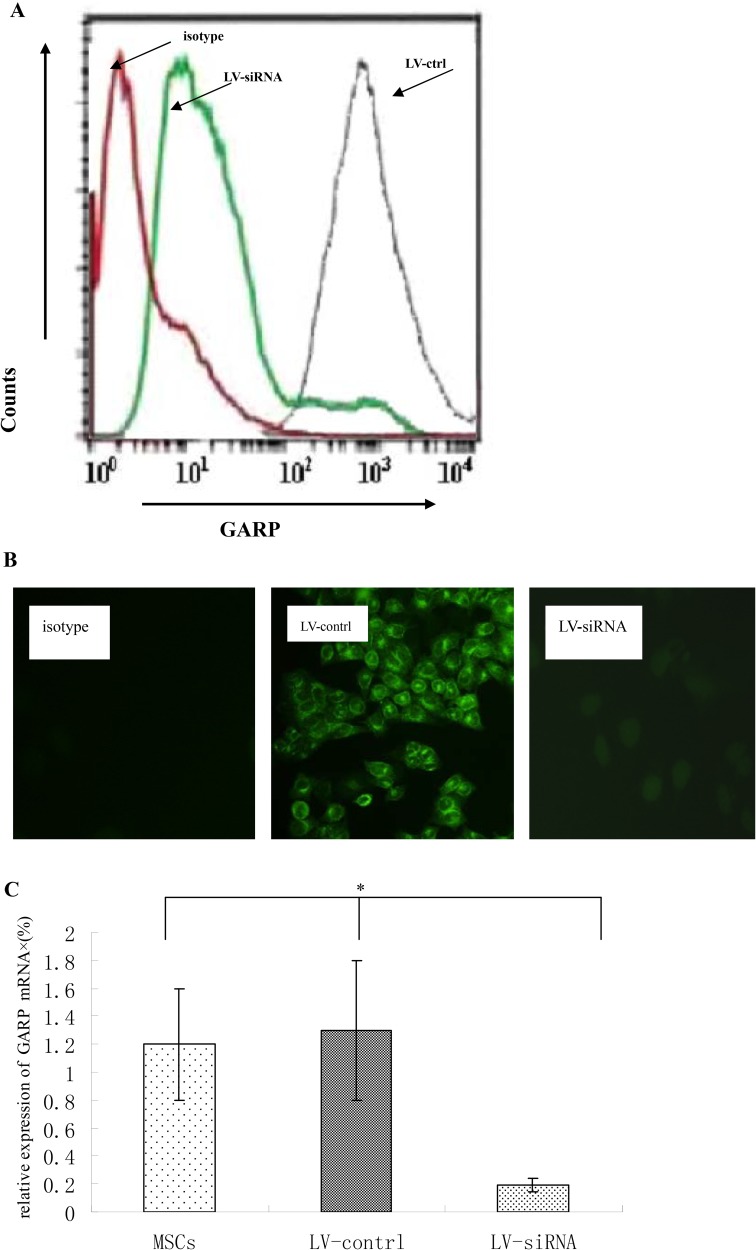
Knocking down GARP expression by lentivial shRNAs Lentiviral particles containing shRNAs targeting GARP transcripts were used to transfect MSCs for 72 h. and then GARP protein levels on MSCs were assessed 72 h later by flowcytometry. (**A**) GARP protein levels were assessed by flowcytometry . Red line indicates isotype control; green line indicates MSCs transfected with LV-siRNA; grey line indicates MSCs transfected with control virus . (**B**) GARP protein levels were assessed by immunofluorescence staining. Original magnification ×200. (**C**) GARP mRNA were assessed by quantitative real-time PCR. ^*^P < 0.05 compared with LV-siRNA. Results are representative of three different experiments.

**Figure 7:**
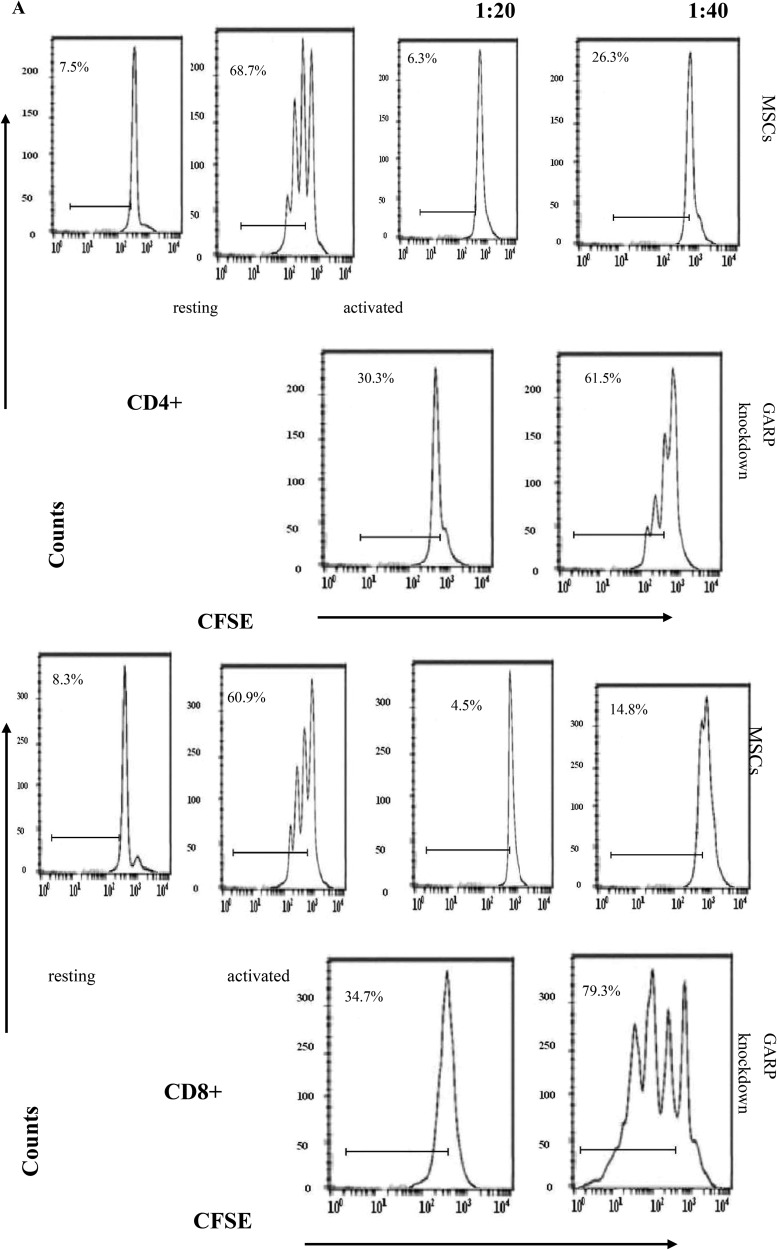

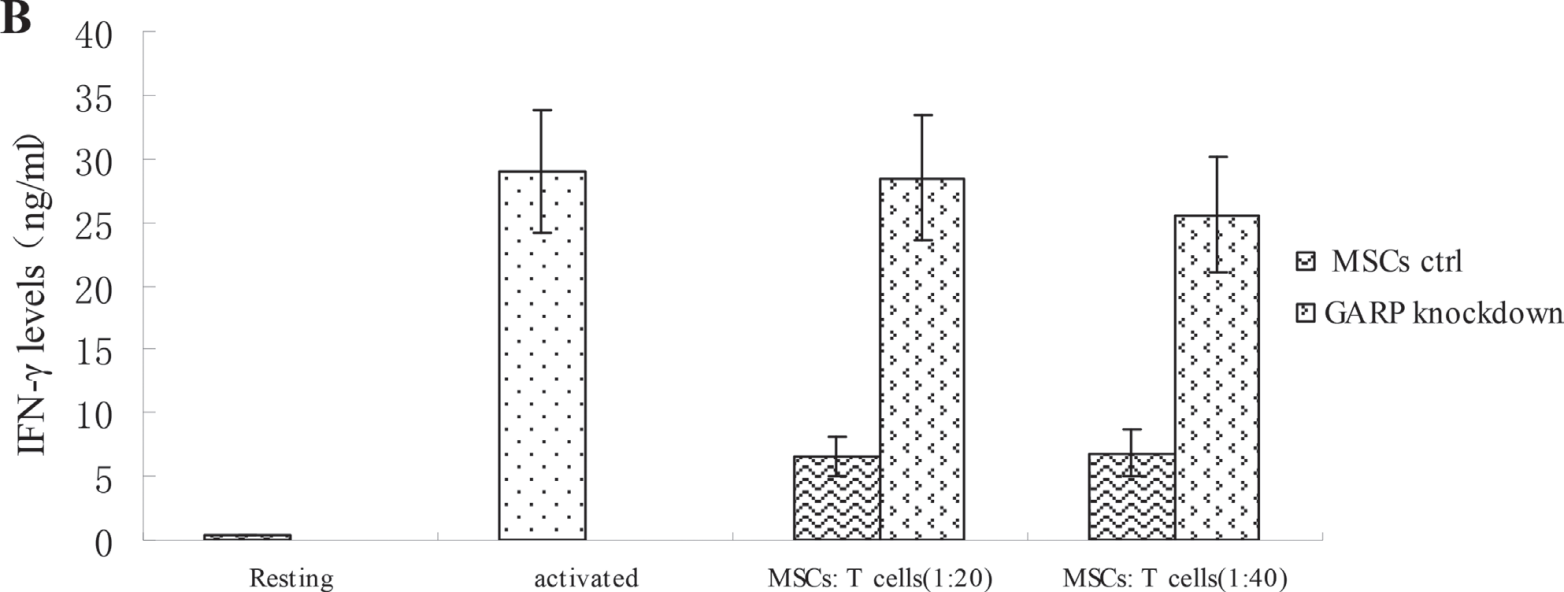
MSCs with GARP knocked down have reduced ability to inhibit T cells WT MSCs were transfected with LV-siRNA or control virus. Seventy-two hours later, these MSCs were cocultured with CFSE-labeled, anti-CD3/CD28 mAb–activated T cells at different ratios for another 72 h. (**A**) Proliferation of the CD4+ and CD8+Tcells was assessed by flow cytometry. (**B**) levels of IFN-γ in the supernatants were measured by standard ELISA. Resting cells are T cells without activation (system negative control); activated cells are T cells incubated with the anti-CD3/CD28 beads in the absence of MSCs (system positive control). Results are representative of three different experiments.

To further confirm the crucial role of GARP in MSCs regarding their T cell inhibitory effect, we applied lentivirus-delivered shRNA to knock down GARP expression on MSCs. We screened the combinations of lentivirus-expressed shRNAs targeting different sites of the GARP transcript by first infecting the cells with the recombinant virus, then assessing GARP mRNA levels in the infected cells 96 h later by flow cytometry and immunofluorescence staining. We identified one combination of viral particles that show most effective to knock down GARP expression on MSCs. It knocked down around 90% of GARP expression in the infected MSCs (Figure 6). The shRNA coding sequence in these two lentiviral particles is 5′-CCGGGATGCTACTCAGGACCTAATCCTCGAGGATTAGGTCCTGAGTAGCATCTTTTTG-3′ or 5′-CCGGATGCCAGCGGTGGAGCAATTACTCGAGTAATTGCTCCACCGCTGGCATTTTTTG-3′, respectively. We then infected MSCs with these selected lentivirus expressed shRNAs after confirming the successful knockdown of GARP expression on the shRNA-infected MSCs 96 h post infection,, we compared their efficacy of inhibiting the proliferation of activated T cells and IFN-γ production from the activated T cells with MSCs infected with the same number of control empty lentivirus particles at the same time. These experiments showed that MSCs with knocked down GARP were less efficient at inhibiting activated T cells than were the control MSCs (Figure 7), confirming that GARP is required for MSCs to inhibit T cells.

### LAP and TGF-β1 co-localized with GARP on MSCs surface

It is reported that cell-to-cell contact is required for MSCs to inhibit T cells [21]. MSCs -produced TGF-β1 is also required for MSCs to efficiently inhibit T cells. This implied that the GARP-activated, MSCs–produced TGF-β1 function in the fashion similar to Tregs [22–23]. In this process, activated TGF-β1 is released.

**Figure 8:**
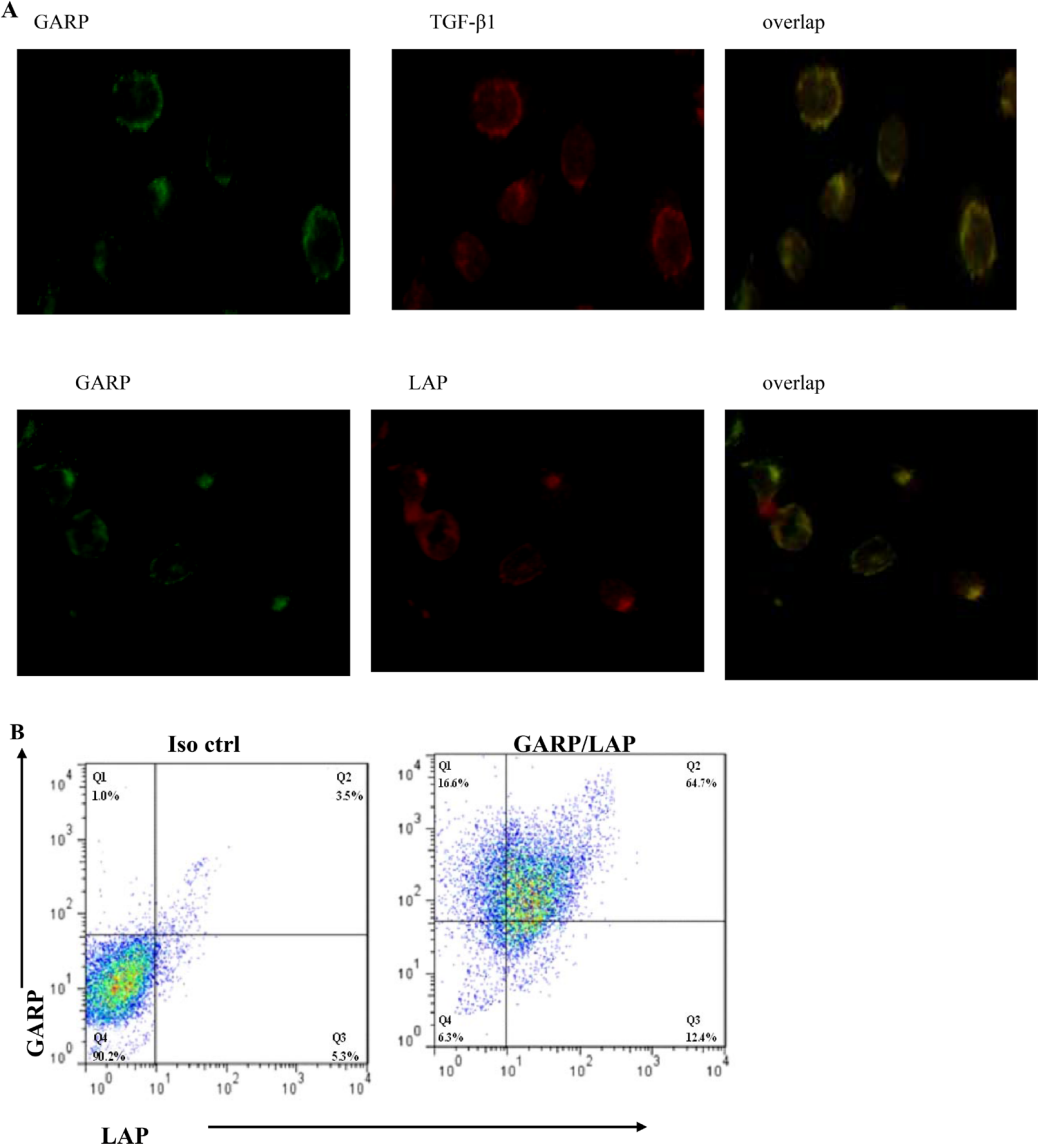

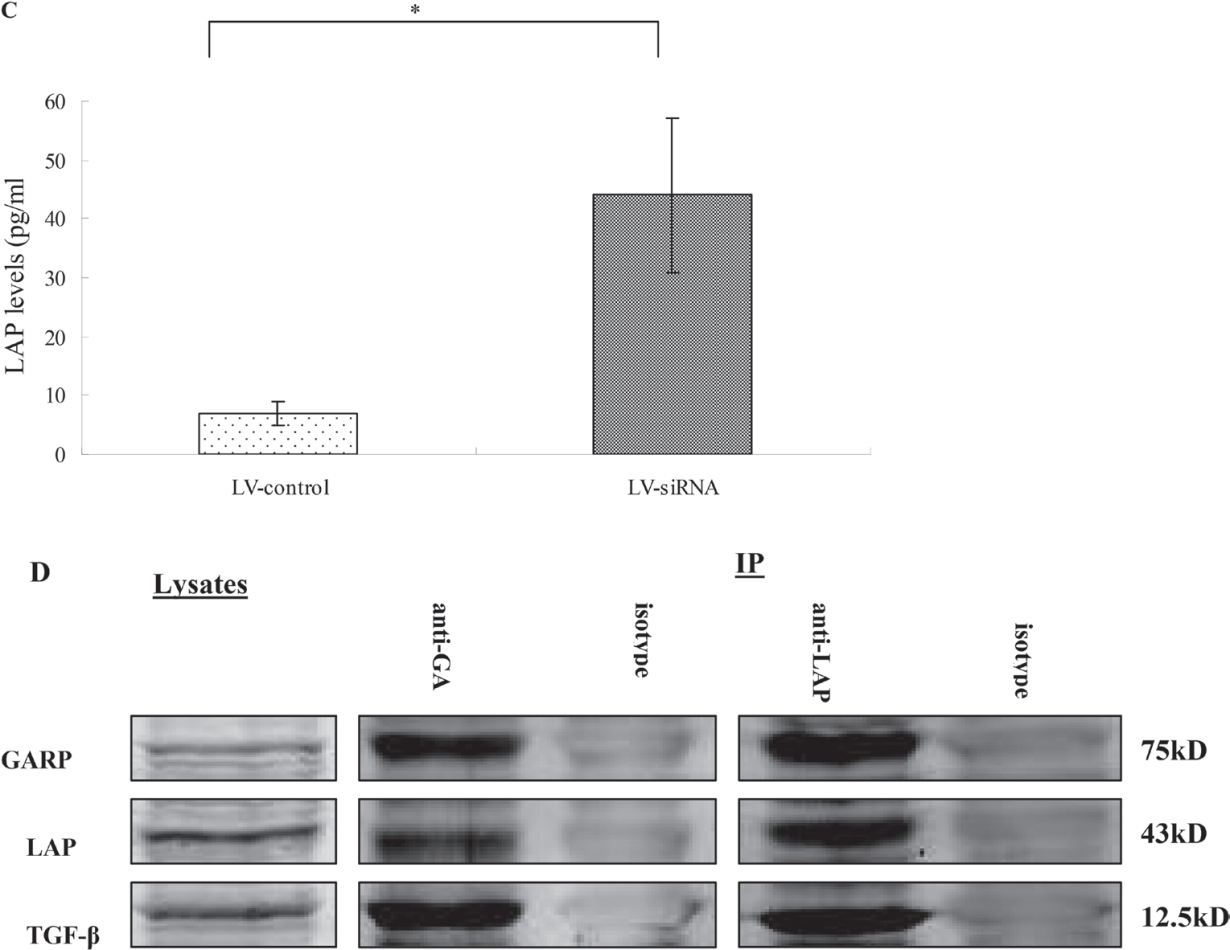
Colocalization of latent TGF-β1 (LAP) and TGF-β1 with GARP on MSCs (**A**) MSCs were cultured on chamber slides and stained with respective Abs against GARP, LAP and TGF-β1. Slides were examined by a fluorescent microscope, and representative images were taken and overlapped to show the colocalization of LAP and TGF-β1 with GARP on MSCs (original magnification ×200). (**B**) MSCs were also double stained with a PE-labeled anti-GARP mAb (clone F011-5) and an allophycocyanin-labeled anti-LAP mAb (clone TW7-16B4) or respective isotype controls, and cells were analyzed by a flow cytometer. (**C**) LAP levels in culture supernatants conditioned by MSCs with GARP knockdown (transfected with LV-siRNA) or by LV-control (transfected with empty virus) were measured by ELISA. ^*^P < 0.05 compared with LV-siRNA. (**D**) Cell lysates and immunoprecipitates were analyzed by Western blot with anti-GARP (top), anti-LAP (middle), and anti-TGF-β1 (bottom) Ab.

To test this hypothesis, we stained MSCs with GARP, LAP and TGF-β1 antibodies, respectively and then examined the cells under the fluorescence microscope. Our data showed that GARP is present on the surface of MSCs. Interestingly, there was also cytoplasmic staining of GARP inside MSCs. Both the LAP and TGF-β1 were present on the surface of MSCs. And their signals colocalized with GARP staining, suggesting that both LAP and TGF-β1 bind to GARP on MSCs (Figure 8A, 8B). Flowcytometric analysis confirmed that MSCs are double positive for both GARP and LAP proteins (Figure 8C). Additionally, we measured levels of LAP by ELISA in the culture supernatants of MSCs with knocked-down GARP. Our data showed that LAP levels of MSCs with knocked-down GARP were significantly higher than the control MSCs, suggesting that GARP function as an important anchor of LAP on MSCs (Figure 8D).

We tested whether GARP binds to LAP by performing co-immunoprecipitation. As shown in Figure 8E, LAP is detected in MSCs lysates that were immunoprecipitated with anti-GARP Ab. Conversely, GARP is detected in a MSCs lysate immunoprecipitated with anti-LAP Ab. The mature TGF-β1 cytokine is also co-immunoprecipitated with GARP (Figure 8E). Since mature TGF-β1 associated with LAP constitutes the latent TGF-β1 molecule, we conclude that latent TGF-β1 binds to GARP.

## DISCUSSION

Using TGF-β1^+/−^ MSCs and TGF-β1 signaling inhibitors, our data showed that MSCs derived TGF-β1 is required for MSCs to repress T cells. Using SMAD3^−/−^ T cells, we showed that MSCs-derived TGF-β1 acts directly on T cells. GARP is constitutively expressed on both human and mouse primary MSCs tested by qPCR, Western blot and flow cytometric analysis.

Using GARP-blocking Abs to neutralize GARP function and GARP-specific shRNAs to knock down GARP expression on MSCs, we proved that GARP is essential to MSCs to efficiently inhibit T cells functions. Additionally, we found that LAP and TGF-β1 colocalize with GARP on MSCs by immunofluorescence staining .

The higher levels of LAP in the culture supernatants were found in MSCs with knocked-down GARP. Taken together, these data show that TGF-β1 is major mechanism by which MSCs inhibit T cells.

GARP is constitutively expressed on MSCs and GARP binds MSCs-produced latent TGF-β1, which is important for the latent TGF-β1 activation. Our results, showing that MSCs inhibit T cells through GARP-associated activation of latent TGF-β1, provide more evidence to support this concept.

MSCs isolated from bone marrow, adipose tissue and umbilical cord tissue have been applied as the main sources for tissue regeneration and their immune regulatory effects have been studied [24–25]. Moreover, MSCs have potential in the immune regulation. Understanding the mechanisms in the process is significant for increasing its effect in treating several autoimmune and inflammatory diseases.

MSCs can secret TGF-β1 that is one of important immunosuppressive cytokines[26]. In this sense, MSCs take advantage of home-grown TGF-β1 to suppress the proliferation of T cells. Interestingly, this immunosuppressive effect could be blocked by anti-TGF-β1 neutralizing antibodies [27].

TGF-β1 produced by MSCs could either alone or in coordination with recruiting Treg cells to generate immunosuppressive effects [28]. Thus, according to several experiment, TGF-β1 is recognized as being responsible for repressive effect of MSCs on CD4^+^ T cells both *in vitro* and *in vivo*. Our data further proved the conclusion that TGF-β1 plays a vital role in MSCs directly inhibiting T cells.

Additionally, the inhibitor SB4531542 or SMAD3^−/−^ T cells, in which the TGF-β1 signaling pathway is impaired, leads to distinctly decreased T cells inhibition by MSCs.

TGF-β1 is produced in a latent form. It is a pivotal and regulatory step for TGF-β1 function and signaling that the mature TGF-β1 is released from the latent TGF-β1 complex.

It has been proved that the mature TGF-β1 is associated with LAP [29–30].Mature TGF-β1 and LAP remain non-covalently bound to each other in a complex called latent TGF-β1. To become active, mature TGF-β1 must be released from the LAP.

The mechanisms of TGF-β1 activation have been described that is the degradation of LAP by proteases or the induction of conformational changes in the latent TGF-β1 [31–32]. But the activation of TGF-β1 by immune cells is not clear.

Recently, the transmembrane protein called GARP was found in human Treg after TCR stimulation [33–35]. The extracellular region of GARP is mainly composed of leucine-rich repeats, motifs present in many proteins that participate in protein–protein interactions [36]. We test the hypothesis that GARP is a receptor for LAP on MSCs.

Our data showed that TGF-β1 and LAP colocalize with GARP on MSCs. We also found that the ability of MSCs inhibiting T cells is correlating with cell-to-cell contact. This finding showed that the MSCs-produced TGF-β1 inhibit T cells is in the similar fashion with Treg.

GARP expression was only reported on platelets and Treg [7–8]. Our data definitely showed that GARP is constitutively expressed on MSCs. This indicates that GARP has the broad distribution and can be the common mechanism of latent TGF-β1 activation.

On the whole, our data indicate that GARP is in fact constitutively present on the surface of MSCs and is increased by IFN-γ. GARP is needed to anchor and activate MSCs-produced latent TGF-β1. Our work has discover the new mechanism by which MSCs suppress immunoreaction.

We suggest GARP as the new marker to identify the activated MSCs and as the target to increase the therapeutic capacity of MSCs.

## MATERIALS AND METHODS

### Mice

All mice used were on a C57BL/6 background. Wild-type (WT), TGF-β1^+/−^ knockout (KO) [37], SMAD3 KO mice [38] and PD-L1^−/−^ mice [39] were purchased from The Jackson Laboratory (Bar Harbor, ME). All the experimental mice were maintained in specific pathogen-free conditions according to the guidelines of the Institute of Laboratory Animal Resources of Xuzhou medical university. All animal experiments were approved by the Institutional Animal Care and Use Committee.

### Isolation of MSCs

Mouse MSCs were isolated from the femur and tibia of WT, TGF-β1^+/−^, and PD-L1^−/−^ mouse following protocols established in the laboratory as described in detail previously [40].

Cells isolated from rat bone marrow were confirmed as MSCs based on their spindle-shaped morphology and adherence to plastic. Surface marker detection of MSCs by flow cytometry showed that MSCs are characteristics of the Sca-1 (+), CD44 (+), CD73 (+), CD90 (−), CD105 (+) and CD34 (−) as previously described [40].

Human primary MSCs were purchased from ScienCell Research Laboratories (Carlsbad, CA).

### Quantitation of mRNA by real-time quantitative PCR

The expression of GARP mRNA was detected by SYBR Green PCR following manufacturer-provided protocols.

Total RNA was prepared by Trizol Reagent (Invitrogen, USA) according to the manufacturer’s instructions. RNA concentration and quality were assessed using spectrophotometer based on the ratio of the absorbance at 260 and 280nm (A260/280). RNA was reverse transcribed at 37°C for 60 minutes followed by 95°C for 5 minutes to inactive the reverse transcriptase. Real-time quantitative PCR was performed using LightCycler 2.0 (Roche Applied Science) in 20 μl volume containing 50 nmol/L of primers,10ng of cDNA, nucleotides, Taq DNA polymerase and the SYBR^®^ Green I. The condition of PCR reaction was: pre-denaturation phase at 94°C for 5 minutes, followed by denaturation at 94°C for 45 seconds; annealing at 58°C for 60 seconds; and extension at 72°C for 60 seconds in totally 40 cycles.

The changes in fluorescence of SYBR Green Ι dye in each cycle were monitored by the software from PE Applied Biosystems. Gene expression was normalized on using beta_actin. Sequence detection primers for GARP were: 5′CAGCGTCGAGAGCAAGTG′(sense); 5′GCTTGGATGTCCAGTGAGAG 3′(anti-sense);

### Western blot of GARP

The protein level of GARP was measured by Western blotting. In brief, the protein lysate was mixed with loading buffer, boiled for 5 mins, and loaded into sodium dodecyl sulfate-polyacrylamide gels (SDS-PAGE) with equal amounts of protein and ran at 200 V for 60 mins followed by transferring to nitrocellulose membranes (Amersham Biosciences) at 100 V for 30 min at room temperature and incubated with primary antibodies. The primary antibodies include GARP antibody (Santa Cruz Biotechnology, CA).The secondary antibodies, anti-goat immunoglobulin G (IgG)-HRP, were purchased from Zhongshan Company (Beijing, China). The protein expression levels were detected by chemiluminescence (ECL system, Amersham, UK) and quantified using Quantity one software (Bio-Rad), the expression level of interested genes was normalized using β-actin.

### GARP knockdown by lentivirus-expressed short hairpin RNAs and MSCs Transduction

To knock down GARP gene expression on MSCs, the shRNA-expressing lentivirus or control empty virus were used to transfect MSCs following previously described protocols [40].

In brief, On the basis of GARP gene sequence (NM:153098.2), use software design shRNA,use BLAST to do the Homologous analysis on Target sequence which had been selected, and to remove nonspecific shRNA. The 21nt fragment selected in GARP gene cDNA sequence was used as the target sequence to synthetize shRNA. The following primers were used:5′ ATCAACGGCCTATTGTCAGGT ’3.

The lentivirus hosting the GARP-shRNA was introduced into the MSCs at a multiplicity of infection of 5 in DMEM for 24 hours with 8 μg/mL polybrene. Infected MSCs were washed twice after 24 hours, and then levels of GARP protein on them were assessed by flow cytometry.

### T cell inhibition assays

T cells from WT or SMAD3 KO mice were enriched using nylonwool columns and labeled with CFSE, then activated with anti–CD3/CD28 mAbs. Different numbers of WT or TGF-β1^+/−^ MSCs were mixed with the activated T cells in the presence of either a polyclonal sheep anti–GARP Ab (R&D Systems, USA). MSCs-mediated T cell inhibition was assessed by measuring the proliferation of both CD4^+^ and CD8^+^ T cells and the production of IFN-γ following protocols described before [40].

### Immunofluorescence assays

Cells were plated in four-well chamber slides, washed with PBS twice, and then fixed for 10 min in fresh 4% paraformaldehyde-PBS. After further washing in PBS, the cells were blocked with 5% BSA for 2 h in PBS. Cells were then washed three more times in PBS with 1% BSA and incubated with primary Ab (anti-GARP polyclonal Ab, anti-mouse LAP, or anti–TGF-β1 polyclonal Ab, Santa Cruz Biotechnology, USA) overnight at 4°C. After washing, cells were incubated with respective secondary Abs at room temperature for 1 h.

The intensity of GARP, LAP or TGF-β1 immunostaining was quantified using a computerized imaging system with 5 randomly selected fields at 200 times magnification. Staining was considered negative when the percentage of cells positive for GARP, LAP or TGF-β1staining was less than 10%.

### ELISAs

LAP, TGF-β1, and INF-γ levels in the culture supernatants were measured by standard ELISAs following manufacturer-provided protocols [40].

LAP, TGF-β1, and INF-γ levels in the culture supernatants were examined by enzyme-linked immunosorbent assay (ELISA), following the manufacturer’s instructions(R&D Systems, USA).The Minimum Detectable Concentrations were 1.6 pg/ml for LAP, 6.7 pg/ml for INF-γ, and 4.61 pg/ml for TGF-β1. Intra-assay and inter-assay coefficients of variation for all ELISA were < 5% and < 10%, respectively. All samples were measured in triplicate.

### Statistical analysis

Experimental data were shown as mean±standard deviation and evaluated using one-way ANOVA for significant differences, differences of *P* < 0.05 were considered statistically significant. Statistical analysis was performed using SPSS12.0 software package.

## Abbreviations

BCA: Bicinchoninic acid
FBS: Fetal bovine serum
PBS: Phosphate buffered saline
PVDF: Polyvinylidene difluoride
HE: Hematoxylin and eosin
MTT: 3-(4,5-dimethylthiazol-2yl)-2,5-diphenyltetrazolium bromide
VEGF: Vascular endothelial growth factor
Flk-1/KDR: fetal liver kinase-1/kinase insert domain containing receptor
MSCs: Mesenchymal stem cells
HUVEC: human umbilical vein endothelial cells
Zvad: carbobenzoxy-valyl-alanyl-aspartyl-[O-methyl]-fluoromethylketone
PCNA: nuclear proliferating cell nuclear antigen
MVD: microvessels density
CM: concentrated conditioned medium
